# Lutein Has a Protective Effect on Hepatotoxicity Induced by Arsenic via Nrf2 Signaling

**DOI:** 10.1155/2015/315205

**Published:** 2015-02-26

**Authors:** Shugang Li, Yusong Ding, Qiang Niu, Shangzhi Xu, Lijuan Pang, Rulin Ma, Mingxia Jing, Gangling Feng, Jing Xia Tang, Qian Zhang, Xiaomei Ma, Yizhong Yan, Jingyu Zhang, Meng Wei, Hai Xia Wang, Feng Li, Shuxia Guo

**Affiliations:** ^1^Department of Public Health and Key Laboratory of Xinjiang Endemic and Ethnic Diseases of the Ministry of Education, Shihezi University School of Medicine, Shihezi 832002, China; ^2^Department of Pathology and Key Laboratory of Xinjiang Endemic and Ethnic Diseases of the Ministry of Education, Shihezi University School of Medicine, Shihezi, Xinjiang 832002, China; ^3^Department of Science and Technology, The Affiliated Tumor Hospital of Xinjiang Medical University, Urumqi 830011, China; ^4^Department of Pathology, The Affiliated Tumor Hospital of Xinjiang Medical University, Urumqi 830011, China

## Abstract

Arsenic produces liver disease through the oxidative stress. While lutein can alleviate cytotoxic and oxidative injury, nuclear factor erythroid 2-related factor 2 (Nrf2) pathway plays a critical role in defending oxidative species. However, the mechanisms by which lutein protects the liver against the effect of arsenic are not known. Therefore, this study aims to investigate the mechanisms involved in the action of lutein using mice model in which hepatotoxicity was induced by arsenic. We found that mice treatment with lutein could reverse changes in morphological and liver indexes and result in a significant improvement in hepatic function comparing with arsenic trioxide group. Lutein treatment improved the activities of antioxidant enzymes and attenuated increasing of ROS and MDA induced by arsenic trioxide. Lutein could increase the mRNA and protein expression of Nrf2 signaling related genes (*Nrf2, Nqo1, Ho-1, and Gst*). These findings provide additional evidence that lutein may be useful for reducing reproductive injury associated with oxidative stress by the activation of Nrf2 signaling. Our findings suggest a possible mechanism of antioxidant lutein in preventing the hepatotoxicity, which implicate that a dietary lutein may be a potential treatment for liver diseases, especially for arsenicosis therapy.

## 1. Introduction

Arsenic has been recognized as a pollutant in drinking water due to its significant toxicity worldwide. The toxicity of arsenic has aroused concern in public health in many countries including China. Using current drinking water standard of 10 *μ*g/L in China, the estimated number of people suffering from arsenic-contaminated water is about 19.6 million people in China [1]. Recent studies have reported the protective effect of vitamin E, selenium, zinc, and so forth [2–4] against arsenic. However, limited data is available for the recommended nutritional intake (RNI) of those substances. Therefore, it is necessary to find a safe nutrient to prevent arsenic toxicity.

At present, the oxidative stress is the most widely accepted mechanism to explain the toxicity induced by arsenic. Liver is the major metabolic organ of the arsenic with the highest concentration of arsenic retention [5]. Consequently, medications with antioxidants are potentially to reduce the hepatotoxicity induced by arsenic. Lutein (LU) is the most common carotenoids in deep yellow vegetables and fruits including cooked spinach, lettuce, string beans, and squash [6]. The biologic properties of lutein and its derivatives have been reported such as being antioxidant neuroprotectant, and studies have indicated the protective role of LU in many diseases including diabetic retinopathy, uveitis, light induced retinopathy, and ischemia/reperfusion injury [7–11].

Nuclear factor erythroid 2-related factor 2 (Nrf2) plays a critical role in defending tissues against elevated oxidative species and toxic damage [12, 13]. In resting cells, the activity of Nrf2 is tightly controlled by Kelch-like ECH-associated protein 1 (Keap1) in the cytoplasm [14]. In response to specific stimuli, keap1 dissociates; then Nrf2 translocates into the nucleus and activates its target genes [15]. This process activates the expression of detoxifying enzymes and antioxidant proteins such as Hmox-1, Nqo1, glutathione transferase (Gst), and glutathione (GSH) [16–19]. Many studies have shown that activation of the Nrf2 pathway could alleviate numerous liver disorders [20–22].

The molecular mechanism of lutein's antioxidation function has not been elucidated. Therefore, the present study aims to assess if lutein can relieve the arsenic-induced hepatotoxicity through activating Nrf2 pathway. If these hypotheses are true, these findings will provide additional evidence that lutein may be useful for reducing reproductive injury associated with oxidative stress by activating Nrf2.

## 2. Materials and Methods

### 2.1. Chemicals

Arsenic trioxide (ATO; As_2_O_3_) was purchased from Beijing Chemical Reagent Corp. (Beijing, China). ATO was dissolved in 1 N NaOH at 0.1 M as a stock solution. LU was obtained from JF-Natural (Tianjin, China). GSH, superoxide dismutase (SOD), total antioxidative capacity (T-AOC), malondialdehyde (MDA), bicinchoninic acid protein, alanine aminotransferase (ALT), and aspartate aminotransferase (AST) assay kits were purchased from Nanjing Jiancheng Bioengineering Institute (Nanjing, China). All other chemicals were of analytical grade and were obtained commercially.

### 2.2. Animals and Treatment

40 healthy Kunming mice (18–22 g, 20 male, 20 female) were obtained from the laboratory center of Xinjiang Medical University (Urumqi, China). The research was approved by the Ethics Committee of Shihezi University. Throughout the experimental period, mice were housed in well-ventilated cages in a temperature-controlled room at 22–25°C. The mice were kept under sterile conditions on a 12 h light/dark cycle. These mice were fed a standard laboratory diet composed of 60% corn meal, 15% beans, 10% bran, 10% corn oil, 3% casein, 1% mineral mixture, and 1% vitamin mixture. The mice had access to food and water ad libitum.

After a two-week acclimation period, the mice were divided based on body weight into four groups: control, ATO-treated, LU-treated, and ATO + LU-treated. Each group consisted of 10 mice (5 males and 5 females) and all groups were dosed intragastrically for five weeks. The control group received normal saline (NS). A previous report showed that both 1 and 5 mg/kg ATO could induce liver injury but did not affect survival in mice [23]. Therefore, the ATO group received 4 mg/kg ATO in our study. The LU group was treated with 40 mg/kg LU, a dose that was shown to reduce the toxic effects of semicarbazide [24]. The ATO + LU group received 4 mg/kg ATO in the morning and 40 mg/kg LU in the evening.

### 2.3. Preparation of Plasma and Liver Function Parameters

The blood samples were collected into evacuated tubes containing heparin solution as anticoagulant and then centrifuged at 3000 g for 10 min. The activities of ALT and AST were detected using commercial kits.

### 2.4. Collection of Liver Tissues and Liver Somatic Index

At the end of the treatment period, the body weight of each mouse was recorded and the mice were sacrificed by cervical dislocation. After washing and drying with filter paper, the livers were collected and weighed to the nearest milligram on an electronic balance (Shimadzu Model BL-220H, Tokyo, Japan). The relative liver weight was calculated according to the following formula: index weight = liver weight/body weight × 100%.

### 2.5. Histopathological Examination of Liver Tissue

After weighing, 3 of liver sections were created. One section was used for preparation of tissue homogenates and one section was frozen in liquid nitrogen and stored at −80°C for total RNA extraction and immunoblot analysis. The remaining sections were fixed in 10% neutral formalin for at least 24 h, dehydrated in different grades of alcohol, and embedded in paraffin. Sections (5 *μ*m) of fixed liver tissue were cut using a rotary microtome. The sections were processed and passed through a graded alcohol series, dyed with hematoxylin and eosin, cleared in xylene, and inspected microscopically as described previously [19].

### 2.6. Preparation of Liver Homogenates

Liver tissues were placed in a lysis buffer (*m*/*v* = 1 : 9) containing 20 mM Tris (pH 7.5), 150 mM NaCl, 1% Triton X-100, and the protein inhibitors sodium pyrophosphate, *β*-glycerophosphate, ethylenediaminetetraacetic acid, Na_3_VO_4_, and leupeptin (Beyotime Biotechnology, Shanghai, China). The liver sections were homogenized with a Tissue Lyser (Qiagen, Valencia, CA, USA). After the homogenate was centrifuged at 2500 ×g for 10 min at 4°C, the supernatant was collected to determine the activity of ALT, AST, SOD, and T-AOC and the content of GSH and MDA.

### 2.7. Measurement of Lipid Peroxidation

MDA, a marker of lipid peroxidation, was measured with a commercial kit following the manufacturer's instructions. Briefly, the samples were treated with thiobarbituric acid, which produces a red compound with an absorption maximum at 532 nm in the presence of MDA. The concentration of MDA was calculated by comparing the absorbance to that produced by the standard, 1,1,3,3-tetraethoxypropane.

### 2.8. Measurement of Antioxidant Enzyme Assay

GSH specifically deoxidizes dithiobisnitrobenzoic acid to form a yellow product, 2-nitro-5-SH-benzoic acid, which can be measured by colorimetry at 532 nm. SOD activity was measured using a tetrazolium salt for detection of superoxide radicals generated by xanthine oxidase and hypoxanthine. One unit of SOD is defined as the amount of enzyme needed to exhibit 50% dismutation of the superoxide radical at 37°C. The reaction product was measured at 450 nm. T-AOC in the tissue was measured with a commercial analysis kit. This kit used antioxidants in the samples to reduce Fe^3+^ to Fe^2+^, which was chelated with porphyrin to produce a purple complex that was quantified by measuring the absorbance at 550 nm. The T-AOC of the samples was determined by comparison with the control standard. Results were normalized to the total amount of protein as measured by bicinchoninic acid protein assay.

### 2.9. Detection of mRNA of Nrf2 Related Genes by Real Time PCR

Liver tissue was snap frozen in liquid nitrogen and total RNA was extracted by Trizol extraction method (Invitrogen, Grand Island, NY, USA) according to the manufacturer's instructions. Equal amounts of RNA (2 *μ*g) were reverse-transcribed into cDNA using the Transcriptor First-Strand cDNA Synthesis Kit (Roche, Indianapolis, IN, USA). Primers were synthesized by Sigma-Aldrich (St. Louis, MO, USA) for the following* Mus musculus* genes:* Hmox-1*; 184 bp amplicon; F, 5′-CAGGTGATGCTGACAGAGGA-3′; R, 5′-ACAGGAAGCTGAGAGTGAGG-3′;* Gst*; 199 bp amplicon; F, 5′-ATCGTTCCCTTTCTCGGCAT-3′; R, 5′-GCAGCCTGTAAGCCATTGAC-3′;* Nqo1*; 112 bp amplicon; F, 5′-TGGCCGAACACAAGAAGCTG-3′; R, 5′-GCTACGAGCACTCTCTCAAACC-3′;* Nrf2*; 173 bp amplicon; F, 5′-TTCCATTTACGGAGACCCAC-3′; R, 5′-ATTCACGCATAGGAGCACTG-3′; and beta-actin; 240 bp amplicon; F, 5′-CACGATGGAGGGGCCGGACTCATC-3′; R, 5′-TAAAGACCTCTATGCCAACACAGT-3′.

The relative gene expression of targets was detected using a comparative cycle threshold method [20]. All samples were tested in triplicate. Real time quantitative PCR (qPCR) was performed on a mixture containing 10 *μ*L of PCR Supermix (Bio-Rad Laboratories, Hercules, CA, USA), 1 *μ*L of forward and reverse primers (Sangon, Beijing, China), 1 *μ*L of template DNA, and 8 *μ*L of distilled water.

The qPCR conditions were as follows: 1 cycle of initial denaturation (94°C for 3 min), 30 cycles of amplification (94°C for 30 s, 57°C for 30 s, and 72°C for 25 s), 1 cycle of melting curve measurement (95°C for 5 s, 65°C for 60 s, and a gradual increase in temperature to 97°C), and a cooling period (40°C for 30 s). The data presented were relative mRNA levels normalized to *β*-actin.

### 2.10. Detection of Protein Expression of Nrf2 Related Protein by Western Blot

Liver tissues were homogenized in one volume of sample buffer (50 mM Tris-Cl, 100 mM DTT, 10% glycerol, and 2% SDS) and centrifuged at 14800 ×g at 4°C for 15 min to move debris. The samples were subjected to SDS-PAGE and transferred to polyvinylidene difluoride membranes. After blocking with skim milk (5%), the blots were probed with the primary antibodies (Abcam, Cambridge, UK) for Nrf2 (1 : 1000), Hmox-1 (1 : 1000), Nqo1 (1 : 1000), Gst (1 : 1000), and *β*-actin (1 : 1000) at 4°Covernight. Incubation with the primary antibodies was followed by incubation with secondary antibodies (conjugated to horseradish peroxidase) after washing in Tris-buffered saline and Tween 20. Blots were processed using an ECL kit (Santa Cruz Biotechnology, Inc.) and exposed to film. All experiments were repeated three times.

### 2.11. Immunohistochemical Staining Examination of Liver Tissue

Paraffin-embedded liver samples were prepared for immunohistochemical analysis to elucidate the subcellular localization of Nrf2 and its target gene products Hmox-1, Nqo1, and Gst. EnVisions two-step immunohistochemical kit (Zhongshan Golden Bridge, Beijing, China) and 3,3′-diaminobenzidine tetrahydrochloride (DAB) enhancer (Dako System, Glostrup, Denmark) were used to detect specific target proteins. Briefly, tissue sections were cut, dried, and deparaffinized. Antigen retrieval was performed by boiling in sodium citrate buffer. After blocking peroxidase and nonspecific binding, tissue sections were incubated with primary and then secondary antibodies (Abcam, Cambridge, UK). The sections were visualized by incubating with DAB and counterstained with hematoxylin.

### 2.12. Statistical Analysis

The results were expressed as the mean ± standard deviation. A general linear model was used to analyze the interactions of the effects of the combination of ATO and LU in a two-factor, two-level factorial design. The model was as follows, in which a significant *P* value for b3 indicated an interaction between the effects of ATO and LU:

(1) $$\begin{array}{c} y=\text{constant}+\text{b1}\times \text{ATO}+\text{b2}\times \text{LU}+\text{b3}\times \left(\text{ATO}\times \text{LU}\right), \end{array}$$

where “*y*” is a measured parameter, such as body weight, liver weight, relative liver weight, liver function, and oxidative stress indices. Analysis of variance (ANOVA) was used to detect the differences among the experimental groups: control, ATO, LU, and ATO + LU. ANOVA was followed by pairwise comparisons with Bonferroni's multiple comparison tests. The data were analyzed using SPSS software for Windows version 15.0 (SPSS Inc., Chicago, IL, USA), and a *P* value < 0.05 was considered to be statistically significant.

## 3. Results

### 3.1. LU Alleviated Liver Damage Induced by ATO in Mice

In our study, we found that the final body weight was significantly lower in ATO group than in the control group (*P* < 0.001). The liver index of the ATO group was higher compared with the control group (*P* < 0.001). We tested the activities of ALT and AST in liver tissue and found both activities of ALT and AST in ATO group were markedly higher than those in the control group (*P* < 0.001). In addition, a significant interaction was found between the effects of ATO and LU on body weight, liver index, and the ALT and AST activities (*P* < 0.001, resp.). ATO + LU treatment increased the body weight and decreased liver index and activities of ALT and AST compared with the ATO group, see Table 1.

**Figure 1:**
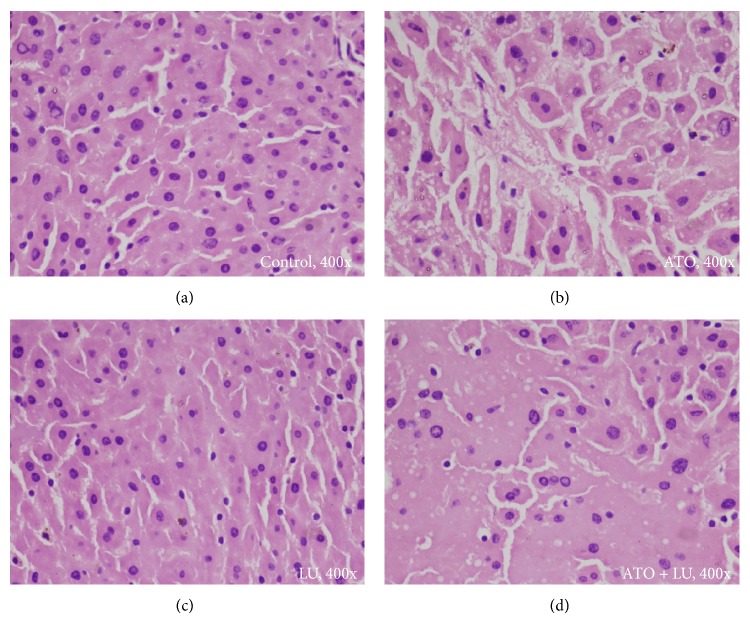
Morphological changes in mouse liver after arsenic trioxide (ATO) and/or lutein (LU). Control group showed normal structure of hepatic cord, hepatic sinusoid, and hepatocyte. The basic performance of the hepatocyte poisoning could be found in ATO treatment group, such as dim boundary of hepatocyte, dismissed cell membrane, cytoplasm disintegrating pieces, and the accumulation of lipid droplets intracytoplasm; meanwhile, the specific hepatocyte balloon degeneration performance can be observed. At the same time, inflammatory cells infiltration scattered in the liver tissue. AOT + LU group displayed fatty degeneration of hepatocytes and congestion but not observed balloon degeneration and hepatocyte disintegration. LU treatment group showed normal structure of liver cells as control group (400x magnification).

To confirm the protective effect of LU on ATO-induced liver damage, we examined liver histology in tissues from the treated mice. LU did not cause noticeable morphological changes in the liver (Figure 1(c)) while arsenic exposure resulted in dim boundary of hepatocyte, dismissed cell membrane, cytoplasm disintegrating pieces, and the accumulation of lipid droplets intracytoplasm, the specific hepatocyte balloon degeneration performance (Figure 1(b)). All these changes were mitigated by LU (Figure 1(d)).

### 3.2. LU Reduced the Oxidative Damage Induced by ATO

We used ATO as an exogenous oxidative stressor in the liver. We tested GSH, SOD, and T-AOC in serum to assess the effects of ATO on endogenous liver antioxidant system. Treatment with ATO caused a prominent decrease of GSH and T-AOC level compared with control group (*P* < 0.01). The content of MDA was significantly higher in ATO group than in control group. There was a statistically significant interaction between ATO and LU on the content of GSH and MDA, as well as the level of T-AOC (*P* < 0.01, resp.). As shown in Table 2, treatment with ATO + LU elevated the content of GSH and the level of T-AOC in liver tissue compared with the ATO group. Additionally, the ATO + LU group had a lower content of MDA than the ATO group. Hence, LU remitted oxidative stress induced by ATO.

### 3.3. LU Activated the mRNA and Protein Expression Levels of Nrf2 Pathway Related Genes

**Figure 2:**
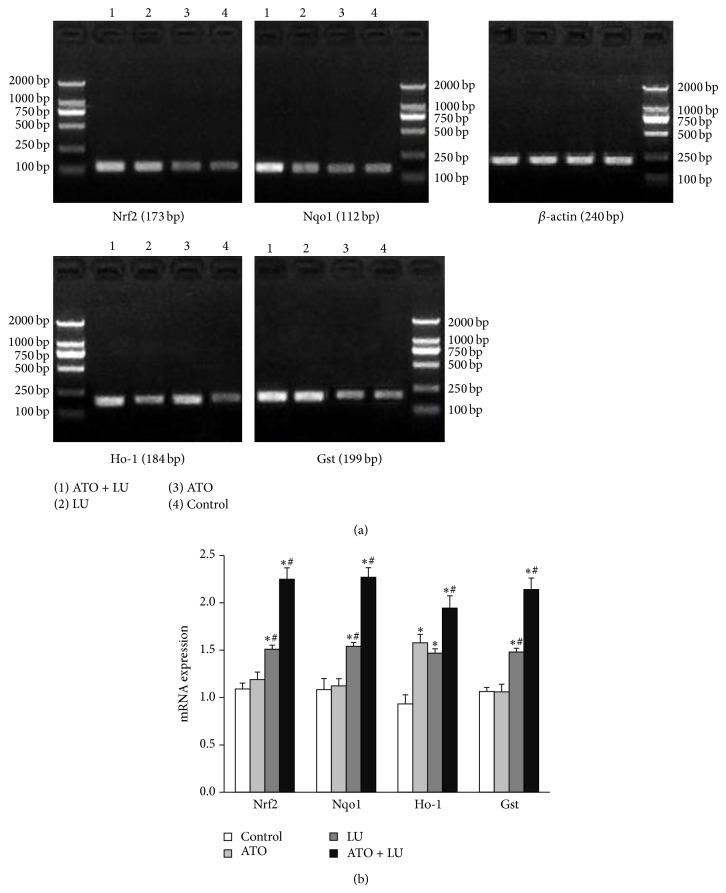
(a) Real time PCR analysis of treatment of arsenic trioxide (ATO) and/or lutein (LU). Nuclear factor erythroid 2-related factor 2 (Nrf2, molecular weight 173 bp), NAD(P)H dehydrogenase, quinone 1 (Nqo1, molecular weight 112 bp), heme oxygenase-1 (Ho-1, molecular weight 184 bp), and glutathione transferase (Gst, molecular weight 199 bp) mRNA expression levels were measured by real time PCR. (b) Quantitative mRNA analysis of treatment of arsenic trioxide (ATO) and/or lutein (LU). *y*-axis indicates mRNA expression of test marker versus *β*-actin. Each bar represents the mean ± SD. The gene expression of Nrf2, Nqo1, Ho-1, and Gst of LU treat group was significantly higher than those of control group. The group treated with ATO + LU showed the most prominent mRNA expression of Nrf2 related genes. Significant differences relative to the control and ATO groups are indicated as follows: ∗ versus control group, *P* < 0.01; # versus ATO group, *P* < 0.01.

**Figure 3:**
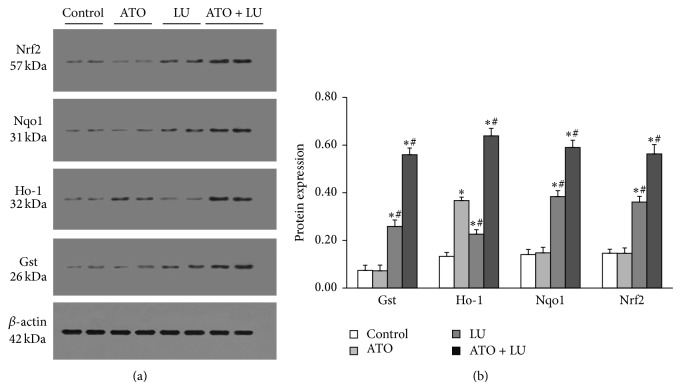
(a) Western blot analysis protein levels of treatment of arsenic trioxide (ATO) and/or lutein (LU). Nrf2 (molecular weight 57 kDa), Nqo1 (molecular weight 31 kDa), Ho-1 (molecular weight 32 kDa), and Gst (molecular weight 26 kDa) protein expression was measured in the livers of mice treated with arsenic trioxide (ATO) and/or lutein (LU) by western blot. (b) Quantitative protein analysis of treatment of arsenic trioxide (ATO) and/or lutein (LU). Nrf2, Nqo1, Ho-1, and Gst protein expression levels were measured. *y* axis represents protein expression of test protein relative to *β*-actin. Each bar represents the mean ± SD. The protein expression of Nrf2, Nqo1, Ho-1, and Gst of LU treat group was significantly higher than those of control group. The group treated with ATO + LU showed the most prominent protein expression of Nrf2 related genes. Significant differences relative to the control and arsenic trioxide (ATO) groups are indicated as follows: ∗ versus control group, *P* < 0.01; # versus ATO group, *P* < 0.01.

We found that neither the mRNA nor the protein expression of Nrf2 in liver was enhanced in ATO group compared with the control group. Similar to Nrf2, expression of its target genes, Nqo1 and Gst were not detectably changed in the ATO group. LU itself induced Nrf2 expression increasingly (*P* < 0.01). The group treated with ATO + LU showed the most prominent expression of Nrf2. Similar to Nrf2, expression of its target genes, Ho-1, Nqo1, and Gst (*P* < 0.01, resp.) were induced in the same two groups (LU and ATO + LU) with the highest level seen in ATO + LU group (Figures 2 and 3).

**Figure 4:**
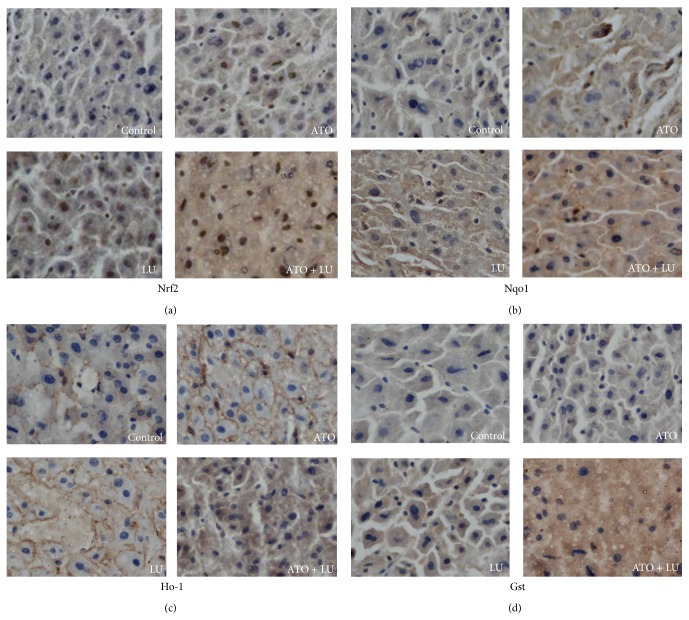
Representative immunohistochemical slides. Immunohistochemical slides were stained for Nrf2, Nqo1, Ho-1, and Gst in liver tissues with arsenic trioxide (ATO) and/or lutein (LU) treatment (200x magnification). Nrf2 protein expression located in nucleus and cytoplasm treated with ATO + LU. Nqo1, Ho-1, and Gst protein expression located in cytoplasm, nucleus, or membrane treated with ATO + LU. The protein expression level of Nrf2, Nqo1, Ho-1, and Gst of ATO + LU group was higher than other groups.

In the present study, we also tested the subcellular localization of Nrf2, Ho-1, Nqo1, and Gst by immunohistochemical staining. We found that both the control and ATO groups showed a low level of Nrf2 in the nucleus while nuclear Nrf2 accumulation was enhanced in the LU group and the ATO + LU group (Figure 4).

## 4. Discussion

Our study shows that LU can relieve arsenic-induced liver dysfunction by activating Nrf2 pathway. Kuitun district (about 4000 Km away from Beijing) next to Shihezi city (in which our university located) is the first arsenic poisoning district in China inner land, founded in 1980. This study provides a good way to protect the hepatotoxicity induced by arsenic, which will help us to prevent and control local arsenic poisoning.

Oxidative stress has been correlated with the progression and severity of many liver diseases. Therefore, antioxidant therapy may be a feasible therapy to reduce liver damage. Arsenic is widely accepted as an exogenous stressor and hepatotoxicant [24]. The major metabolic pathway of arsenic is methylation in the liver [25]. However, the methylation of arsenic is not a detoxification process because trivalent methylated arsenical intermediates are highly toxic [26]. In our study, we used arsenic to induce oxidative stress and liver injury in mice. The toxic effects of arsenic are related to its ability to induce reactive oxygen species [25]. After 35-day treatment with ATO, we found that ATO inhibited the overall growth in mice and liver injury as measured by the increased level of tissue ALT and AST compared with control group. Further, noticeable morphological changes found in the liver further confirmed the adverse effects of ATO on the liver. Compared with the control group, livers from mice treated with ATO showed significantly decreased GSH content and T-AOC level and increased MDA content. These results strongly suggested that ATO disrupted the balance between oxidant and antioxidant agents and demonstrated ATO-induced liver oxidative damage.

One large scale randomized placebo-controlled trial sponsored by NIH lutein demonstrated that LU was effective in decreasing the risk of developing advanced age-related macular degeneration (AMD). To the best of our knowledge, there have been no reports on the effects of lutein on neurons in the brain in a rodent stroke model [27]. In the present study, we found significant interactions between LU and ATO treatments on body weight, liver index, ALT, AST, T-AOC levels, GSH, and MDA contents. We observed that these parameters in the ATO + LU group fell in between the ATO and control group. This indicates that LU can alleviate the oxidative stress and liver injury induced by ATO. Compared to the ATO group, pathological improvement in ATO + LU group provided evidence that LU antagonizes the hepatotoxicity of ATO.

Previous studies have shown that LU could reduce oxidative damage and improve the liver function. However, few studies addressed the exact mechanisms of these protective effects and the involvement of Nrf2 pathway. Activation of Nrf2 pathway can enhance the expression of detoxifying enzymes and antioxidants [15, 17] and attenuate hepatic fibrosis [18], nonalcoholic steatohepatitis [20], NAFLD [28], and drug-induced liver injury [21]. In our study, we found that LU can induce the expression of Nrf2 compared with control group. In addition, the expression of Nrf2 was significantly highest in ATO + LU group. We also measured the target genes of Nrf2, such as* Hmox-1*,* Nqo1*, and* Gs*t in the present study. Hmox-1 possesses cytoprotective properties by promoting the oxidative cleavage of the prooxidant heme to carbon monoxide and bilirubin [28]. Nqo1 is a phase II enzyme and could reduce oxidative stress through reducing superoxide, maintaining endogenous antioxidants and catalyzing the metabolism of xenobiotics [28]. Gst is responsible for catalyzing the conjugation of GSH to reactive electrophiles [29]. Similar to Nrf2, expressions of Hmox-1, Nqo1, and Gst were all induced in the two LU-treated groups with the highest levels seen in ATO + LU group at both the mRNA and protein levels. In addition, LU also increased the liver content of GSH and the level of T-AOC, decreased the liver content MDA, and alleviated the pathological alterations induced by ATO. Based on the alteration of oxidative stress parameters, mitigation of pathological changes, and enhanced expression of Nrf2 pathway components, we concluded that LU stimulated the Nrf2 pathway, thus alleviating oxidative stress and reducing oxidative damage in the liver.

Numerous studies have shown that arsenic was an Nrf2 inducer in several cell types, including human hepatocytes [30–32]. It is very interesting that the expressions of either Nrf2 or its downstream genes,* Noq1* and* Gst*, were enhanced in the ATO group in our study. Recent studies have shown that expression levels of Nrf2 and its target genes initially increased and then gradually fell over the duration of arsenic exposure [33]. Acute exposure to arsenic or other exogenous stressors may activate the Nrf2 pathway to maintain cellular redox homeostasis and limit oxidative damage, but the protective compensatory reaction may be overpowered as exposure time may be prolonged. It has been shown that Nrf2 is repressed by Keap1 in the cytosol and degraded by the 26S proteasome in nonstressed cells. Oxidative stress activates Nrf2 by permitting its dissociation from Keap1 and translocation into the nucleus where it binds to the antioxidant response element and leads to the expression of the target genes. Given that subcellular localization is a major determining factor of the function of Nrf2, we studied its subcellular location by immunohistochemical staining. We found prominent nuclear accumulation of Nrf2 protein in both the LU and ATO + LU groups. Meanwhile, Hmox-1, Nqo1, and GST also showed accumulation in the nucleus.

In summary, we demonstrated that LU could alleviate arsenic-induced oxidative stress and liver injury through activating the Nrf2 pathway. As oxidative damage is an important toxic mechanism of arsenic and is associated with the pathological processes of diverse liver diseases, we believe LU could be useful in treating many liver disorders through reducing oxidative stress by activating Nrf2 pathway. We also found the possible role of the Nrf2 pathway in prolonged oxidative stress. Our findings could help provide a better understanding of the mechanism of LU. This study also provided more evidence that a dietary Nrf2 activator might be a plausible treatment for liver diseases. Further studies to clarify these findings should include validation of the role of the Nrf2 pathway by studying the effects of LU on liver injury in mice lacking the Nrf2 gene and cell experiments such as the dissociation of Keap1/Nrf2 complex, the translocation of Nrf2, and the accumulation of Nrf2 in nucleus.

## Figures and Tables

**Table 1:** Effect of arsenic trioxide (ATO) and/or lutein (LU) administration on indices related to hepatotoxicity in mice.

	Control (NS)	ATO (4 mg/kg)	LU (40 mg/Kg)	ATO + LU (4 mg/kg + 40 mg/Kg)
Initial body weight (g)	19.41 ± 1.16	19.31 ± 1.69	21.12 ± 1.43	20.50 ± 1.59
Final body weight (g)	34.68 ± 2.03	34.82 ± 1.19^ac^	25.83 ± 1.96	30.37 ± 1.91^ab^
Body weight gain (g)	15.27 ± 2.65	15.52 ± 1.71	4.71 ± 2.68	9.88 ± 1.93
Liver weight (g)	1.38 ± 0.17	3.23 ± 0.15^ab^	1.05 ± 0.07^a^	1.16 ± 0.26^ab^
Liver index (%)	3.99 ± 0.61	9.29 ± 0.47^b^	4.1 ± 0.49^a^	3.88 ± 0.96^b^
AST (U/g prot)	22.01 ± 4.95	31.62 ± 7.28^b^	17.77 ± 4.97^a^	10.87 ± 4.94^ab^
ALT (U/g prot)	78.34 ± 21.40	136.51 ± 29.31^b^	68.84 ± 17.25^a^	59.98 ± 17.08^b^

Note: the results were described as mean ± SD (*n* = 10). ^a^Indicating significant difference from control (NS) group at *P* < 0.01; ^b^indicating significant difference from the ATO group (4 mg/kg) at *P* < 0.01; ^c^indicating significant difference from the ATO + LU group (4 mg/kg + 40 mg/Kg) at *P* < 0.01.

**Table 2:** Effect of lutein (LU) on malondialdehyde (MDA), glutathione (GSH), superoxide dismutase (SOD), and total antioxidative capacity (T-AOC) of arsenic trioxide- (ATO-) treated mice.

Experimental group	MDA nmol/mg prot	GSH *μ*mol/g prot	SOD U/g prot	T-AOC U/g prot
Control (NS)	2.89 ± 0.45	0.61 ± 0.04	0.48 ± 0.18	37.19 ± 5.79
LU (40 mg/kg)	3.03 ± 0.46	0.77 ± 0.06	0.56 ± 0.17	46.57 ± 2.21^b^
ATO (4 mg/kg)	4.73 ± 0.54^ac^	0.50 ± 0.05^ac^	0.54 ± 0.14^ac^	27.28 ± 4.13^ac^
ATO + LU (4 mg/kg + 40 mg/kg)	3.58 ± 0.33^ab^	0.67 ± 0.02^ab^	0.55 ± 0.17^ab^	29.38 ± 3.20^ab^

Note: the results were described as mean ± SD (*n* = 10). ^a^Indicating significant difference from control (NS) group at *P* < 0.01; ^b^indicating significant difference from the ATO group (4 mg/kg) at *P* < 0.01; ^c^indicating significant difference from the ATO + LU group (4 mg/kg + 40 mg/Kg) at *P* < 0.01.
